# Porcine Deltacoronavirus, Thailand, 2015

**DOI:** 10.3201/eid2204.151852

**Published:** 2016-04

**Authors:** Taveesak Janetanakit, Mongkol Lumyai, Napawan Bunpapong, Supanat Boonyapisitsopa, Supassama Chaiyawong, Nutthawan Nonthabenjawan, Sawang Kesdaengsakonwut, Alongkorn Amonsin

**Affiliations:** Chulalongkorn University, Bangkok, Thailand

**Keywords:** porcine deltacoronavirus, coronavirus, viruses, pigs, Thailand

**To the Editor:** Porcine deltacoronavirus (PDCoV) was first reported in Hong Kong in 2012 and included the HKU15-44 and HKU15-155 strains (*1*). In early 2014, PDCoV was reported in pigs with diarrhea on swine farms in Ohio, USA (*2*), and later in other states (*2*–*5*). In April 2014, PDCoV strain KNU14-04 was reported in pigs in South Korea (*6*). A retrospective study in 2012 reported PDCoV strain S27 in Sichuan, China (*7*). Recently PDCoV strain CNJXNI2 has been reported in pigs with diarrhea in Jiangxi, China (*8*).

There are currently 28 complete PDCoV genomes from China, South Korea, and the United States available in GenBank. We report emergence of PDCoV infections on a commercial swine farm in Thailand.

In June 2015, we investigated reports of acute diarrhea in piglets, gilts, and sows on a swine farm. An outbreak occurred on a commercial swine farm (3,000 sows) located in the eastern province of Thailand. Clinical signs, including acute watery diarrhea, loss of appetite, and agalactia, were observed in gilts and sows in the breeding and gestation houses. Subsequently, piglets in farrowing houses had clinical signs (depression, fever, watery diarrhea, and severe dehydration). Although clinical signs were detected less frequently in fattening pigs in growth-finishing houses, PDCoVs were later detected from blood samples of fattening pigs.

The outbreak lasted 6 weeks (June 10–July 20, 2015). The mortality rate was 27.63% (829/3,000) in sows and 64.27% (2,892/4,500) in piglets but was lower than that usually observed for porcine epidemic diarrhea virus (PEDV) infection. A total of 865 (19.22%) piglets died and were culled during 10 production weeks. Postmortem examination of dead piglets showed emaciated animals and yellow pasty feces. Intestines and colons showed thin walls with a watery content and curdled milk. Histopathologic examination showed shortened and fused villi in the jejunum and ileum. An attenuated and vacuolated cytoplasm in enterocytes was also observed (Technical Appendix Figure 1) (*9*,*10*).

We examined 30 samples from the affected swine farm. Blood (n = 10), intestine (n = 8), lymph node (n = 2), feces (n = 6), and feed (n = 4) samples were collected for 2 day-old piglets and 17-, 19-, and 20-week-old fattening pigs. A total of 26 samples were positive for PDCoV by reverse transcription PCR (*2*) (Technical Appendix Table 1). Because sick pigs had clinical signs similar to those of pigs with other swine virus diseases, all samples were tested for transmissible gastroenteritis coronavirus; PEDV; rotaviruses A, B, and C; porcine reproductive and respiratory syndrome virus; and circovirus. All test results were negative.

We selected 2 PDCoVs (S5011 and S5015L) for whole-genome sequencing and 14 PDCoVs for sequencing of spike (S), envelope (E), membrane (M), and nucleocapsid (N) genes and the 3′-untranslated region (UTR). Nucleotide sequences obtained were submitted to GenBank (Technical Appendix Table 2).

Sequence analysis of the 2 PDCoVs from Thailand showed that their whole genomes had 99.98% nt identity (only 4 nt differences) with each other and highest nucleotide identities with PDCoVs from China (98.43% with AH2004). S gene sequences showed greatest diversity (99.97%–100% nt identities and 99.91%–100% aa identities) for PDCoVs from Thailand and 95.93%–96.68% with other reference PDCoVs, which is consistent with findings of previous report (*5*). In contrast, E, M, and N genes were conserved (100% nt identities for PDCoVs from Thailand and 99.19%–100% for E genes, 98.28%–99.07% for M genes, and 96.88%–97.81% for N genes with reference PDCoVs) (Technical Appendix Table 3).

Phylogenetic analysis of the whole genome of PDCoVs from Thailand showed close relatedness with AH-2004, HKU15-44, S27-2012, and HKU15-155 virus strains from China. However, these viruses from Thailand were in a different subcluster than PDCoVs from the United States (Figure; Technical Appendix Figure 2). PDCoVs identified in this study might represent a new variant of PDCoV because these 2 viruses have unique sequence characteristics: 3-nt (TCT) and 1-nt (A) deletions in the 5′-UTR, 6-nt (AGTTTG) and 9-nt (GAGCCAGTC) deletions in open reading frame 1a/b, and 4-nt (CTCT) insertion in the 3′-UTR (Technical Appendix Table 4).

**Figure:**
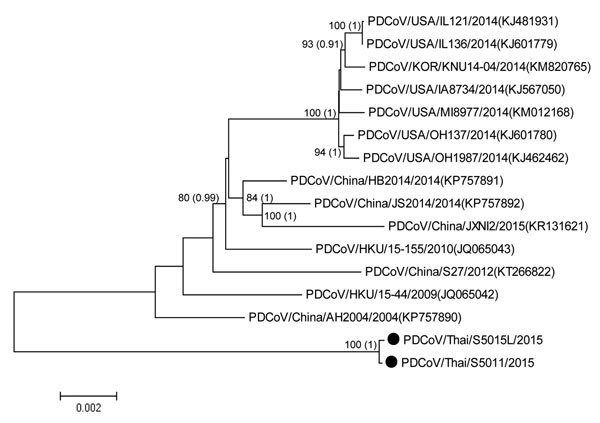
Phylogenetic analysis of whole-genome sequences of porcine deltacoronaviruses (PDCoVs), Thailand. Black circles indicate strains isolated in this study. The tree was constructed by using MEGA version 6.06 (http://www.megasoftware.net/) with the neighbor-joining algorithm and bootstrap analysis with 1,000 replications and BEAST (http://beast.bio.ed.ac.uk/) with Bayesian Markov chain Monte Carlo analysis of 5,000,000 generations and an average SD of split frequencies <0.05. Numbers along branches are bootstrap values (posterior probabilities). Scale bar indicates nucleotide substitutions per site.

We identified PDCoV on a commercial swine farm in Thailand. Affected pigs had clinical signs of acute watery diarrhea, similar to those of pigs infected with PEDV, and had moderate illness and low mortality rates. PDCoVs were detected in symptomatic piglets, sows, and fattening pigs, although clinical signs in fattening pigs were least severe.

Swine farmers and veterinarians should be aware of PDCoV as another causative agent of watery diarrhea in pigs. Similar to PEDV, Wang et al. reported that sequence deletions, insertions, and mutations in PDCoVs in pigs might contribute to variant virus virulence (*2*).

Our findings might assist in development of diagnostic assays for differentiating PDCoVs in Thailand from PDCoVs in other countries. Because PDCoVs from Thailand were highly related to each other, PDCoV might have transmitted into Thailand by a single event. However, verification of this possibility would be difficult. Similar to the situation in the United States, PDCoV might be underdiagnosed in Thailand.

Technical Appendix Methods used and additional information for detection of porcine deltacoronavirus, Thailand, 2015.
