# Supplementary material for: Porcine Deltacoronavirus, Thailand, 2015
Source: Emerg Infect Dis. 2016 Apr;22(4):757–9. doi: 10.3201/eid2204.151852 (PMC4806967; doi:10.3201/eid2204.151852)
Supplement: Technical Appendix — Methods used and additional information for detection of porcine deltacoronavirus, Thailand, 2015. [file 15-1852-Techapp-s1.pdf]

Article DOI: http://dx.doi.org/10.3201/eid2204.151852

Porcine Deltacoronavirus, Thailand, 2015

Technical Appendix

Porcine Deltacoronavirus Outbreak Investigation

During June–July 2015, the Center of Excellence for Emerging and Re-emerging Diseases in Animals at Chulalongkorn University (Bangkok, Thailand) investigated a suspected outbreak of porcine deltacoronavirus (PDCoV) infection in piglets with acute diarrhea, gilts, and sows that occurred on a commercial swine farm. Epidemiolgic investigation, pathologic examination, sample collection, and laboratory diagnosis were conducted to determine the cause of the outbreak.

Identification of Porcine Deltacoronavirus

Blood (n = 10), intestine (n = 8), lymph node (n = 2), feces (n = 6), and feed (n = 4) samples were collected for 2 day-old piglets and 17-, 19-, and 20-week-old fattening pigs. Because sick pigs had clinical signs similar to those of pigs with other swine virus diseases, all samples were tested for transmissible gastroenteritis coronavirus; PEDV; rotaviruses A, B, and C; porcine reproductive and respiratory syndrome virus, and circovirus (*1*–*5*). For PDCoV identification, RNA was extracted from homogenized tissue and fecal, blood, and feed samples by using the QIAamp RNA Mini Kit (QIAGEN, Hilden, Germany) according to the manufacturer’s instructions.

cDNA was synthesized by using the Improm-II Reverse Transcription System and random primer (Promega, Madison, WI, USA). In brief, 5 µL of virus RNA and 5 µL of random primers (100 µmol/L) in a total reaction volume of 22 µL were incubated at 70°C for 15 min and at 4°C for 5 minutes. A total of 4 µL of 5× cDNA buffer, 1 µL of 0.5 mmol/L dNTP mixture, 2 µL of 2.5 mmol/L $MgCl_2$, 0.3 µL of RNase inhibitor, 1 µL of ImProm-II reverse transcriptase, and 3.7 µL of distilled water were added to the RNA–primer mixture. The mixture was incubated at 25°C for 5 min, 42°C for 60 min, and 72°C for 15 min.

PDCoV identification was conducted by using a PCR protocol previously described (*6*). In brief, 10 µL of PCR mixture contained 0.5 µL of cDNA, 0.4 µL (10 µmol/L) of each forward and reverse primer, 5 µL of 2× TOP Taq Master Mix (QIAGEN), 1 µL of 10× CoralLoad, and 2.7 µL of distilled water. PCR conditions for PDCoV identification were initial denaturation at 94°C for 3 min;

40 cycles of denaturation at 94°C for 30 s, annealing at 50°C for 45 s, and extension at 72°C for 1 min; and final extension at 72°C for 7 min.

PCR products were then visualized by gel electrophoresis on a 1.2% of agarose gel in 0.5× Tris borate EDTA). Expected PDCoV product sizes were 500 bp for the membrane gene and 700 bp for the nucleocapsid gene.

Characterization of Porcine Deltacoronavirus

We selected 2 PDCoVs (S5011 and S5015L) for whole-genome sequencing and 14 PDCoVs for sequencing of spike, envelope, membrane, and nucleocapsid genes and the 3′–untranslated region. PDCoV genomes were amplified by using PCR and oligonucleotide primer sets previously described or new primer sets designed by using the Primer3 program (*6*–*8*). Primer sequences are available upon request.

A total of 30 μL of PCR mixture contained 2 μL of cDNA, 1.2 μL (10 μmol/L) of each forward and reverse primer, 15 μL of 2× TOPTaq Master Mix (QIAGEN), 3 μL of 10× CoralLoad, and 8.1 μL of distilled water. PCR conditions were initial denaturation at 94°C for 3 min; 40 cycles of denaturation at 94°C for 30 s, annealing at 48°C for 45 s, and extension at 72°C for 2 min; and final extension at 72°C for 7 min. Amplicons were gel-purified and sequenced (1st Base Laboratories, Kembangan, Malaysia).

Nucleotide sequences were assembled and validated by using SeqMan software version 5.03 (DNASTAR Inc., Madison, WI, USA). Nucleotide sequences of PDCoVs from Thailand were submitted to GenBank under accession nos. KU-51641–KU051656.

For pairwise comparison and genetic analysis of PDCoVs, nucleotide sequences and deduced amino acids of PDCoVs from Thailand were aligned with those of reference PDCoVs from China, South Korea, and the United States by MEGA version 6.06 and MegAlign version 5.03 (DNASTAR Inc., Madison, WI, USA) software. For phylogenetic analysis, whole-genome sequences of PDCoVs from Thailand were compared with those of reference PDCoVs. Phylogenetic analysis was performed by using MEGA version 6.06 (http://www.megasoftware.net/) with the neighbor-joining algorithm and bootstrap analysis of 1,000 replications. Additional analysis was performed by using BEAST software (http://beast.bio.ed.ac.uk/) and Bayesian Markov chain Monte Carlo methods with 5,000,000 generations and an average SD of split frequencies <0.05 (*9*–*11*).

References

1. Alfieri AA, Leite JP, Alfieri AF, Jiang B, Glass RI, Gentsch JR. Detection of field isolates of human and animal group C rotavirus by reverse transcription-polymerase chain reaction and digoxigenin-labeled oligonucleotide probes. J Virol Methods. 1999;83:35–43. PubMed http://dx.doi.org/10.1016/S0166-0934(99)00104-4

2. Chen JF, Sun DB, Wang CB, Shi HY, Cui XC, Liu SW, et al. Molecular characterization and phylogenetic analysis of membrane protein genes of porcine epidemic diarrhea virus isolates in China. Virus Genes. 2008;36:355–64. PubMed http://dx.doi.org/10.1007/s11262-007-0196-7

3. Gouvea V, Allen JR, Glass RI, Fang ZY, Bremont M, Cohen J, et al. Detection of group B and C rotaviruses by polymerase chain reaction. J Clin Microbiol. 1991;29:519–23. PubMed

4. Nguyen VG, Moon HJ, Park SJ, Kim HK, Keum HO, Rho SM, et al. Multiplex PCR for PRRSV detection and typing. In: D'Allaire S, Friendship R, editors. 21st International Pig Veterinary Society Congress, July 18–21, 2010. Vancouver, British Columbia, Canada; 2010. Perry (IA): American Association of Swine Veterinarians [cited 2016 Jan 5]. https://www.aasv.org/library/swineinfo/series_index.php?id=9#82

5. Song DS, Kang BK, Oh JS, Ha GW, Yang JS, Moon HJ, et al. Multiplex reverse transcription-PCR for rapid differential detection of porcine epidemic diarrhea virus, transmissible gastroenteritis virus, and porcine group A rotavirus. J Vet Diagn Invest. 2006;18:278–81. PubMed http://dx.doi.org/10.1177/104063870601800309

6. Wang L, Byrum B, Zhang Y. Detection and genetic characterization of deltacoronavirus in pigs, Ohio, USA, 2014. Emerg Infect Dis. 2014;20:1227–30. PubMed http://dx.doi.org/10.3201/eid2007.140296

7. Koressaar T, Remm M. Enhancements and modifications of primer design program Primer3. Bioinformatics. 2007;23:1289–91. PubMed http://dx.doi.org/10.1093/bioinformatics/btm091

8. Untergasser A, Cutcutache I, Koressaar T, Ye J, Faircloth BC, Remm M, et al. Primer3: new capabilities and interfaces. Nucleic Acids Res. 2012;40:e115. PubMed http://dx.doi.org/10.1093/nar/gks596

9. Drummond AJ, Rambaut A. BEAST: Bayesian evolutionary analysis by sampling trees. BMC Evol Biol. 2007;7:214. PubMed http://dx.doi.org/10.1186/1471-2148-7-214

10. Drummond AJ, Suchard MA, Xie D, Rambaut A. Bayesian phylogenetics with BEAUti and the BEAST 1.7. Mol Biol Evol. 2012;29:1969–73. PubMed http://dx.doi.org/10.1093/molbev/mss075

11. Tamura K, Dudley J, Nei M, Kumar S. MEGA4: Molecular Evolutionary Genetics Analysis (MEGA) software version 4.0. Mol Biol Evol. 2007;24:1596–9. PubMed http://dx.doi.org/10.1093/molbev/msm092

12. Woo PC, Lau SK, Lam CS, Lau CC, Tsang AK, Lau JH, et al. Discovery of seven novel mammalian and avian coronaviruses in the genus deltacoronavirus supports bat coronaviruses as the gene source of alphacoronavirus and betacoronavirus and avian coronaviruses as the gene source of gammacoronavirus and deltacoronavirus. J Virol. 2012;86:3995–4008. PubMed http://dx.doi.org/10.1128/JVI.06540-11

13. Wang YW, Yue H, Fang W, Huang YW. Complete genome sequence of porcine deltacoronavirus strain CH/Sichuan/S27/2012 from mainland China. Genome Announc. 2015;3:e00945–15. PubMed

14. Song D, Zhou X, Peng Q, Chen Y, Zhang F, Huang T, et al. Newly emerged porcine deltacoronavirus associated with diarrhoea in swine in China: identification, prevalence and full-length genome sequence analysis. Transbound Emerg Dis. 2015;62:575–80. PubMed

15. Li G, Chen Q, Harmon KM, Yoon KJ, Schwartz KJ, Hoogland MJ, et al. Full-length genome sequence of porcine deltacoronavirus strain USA/IA/2014/8734. Genome Announc. 2014;2:e00278–14. PubMed http://dx.doi.org/10.1128/genomeA.00278-14

16. Lee S, Lee C. Complete genome characterization of Korean porcine deltacoronavirus strain KOR/KNU14–04/2014. Genome Announc. 2014;2:e01191–14. PubMed http://dx.doi.org/10.1128/genomeA.01191-14

Technical Appendix Table 1. Characteristics of pig samples examined for porcine deltacoronavirus and other viruses, Thailand, 2015*

Sample ID	Date of collection	Pig age	Sample	PDCoV	PEDV	TGEV	RVA	RVB	RVC	PPRSV	Circovirus
S5011	Jun 10	2 d	Intestine	+	–	–	–	–	–	–	–
S5012	Jun 10	2 d	Intestine	+	–	–	–	–	–	–	–
S5013	Jun 10	NA	Feces	+	–	–	–	–	–	–	–
S5014P	Jun 30	2 d	GI pool	+	–	–	–	–	–	–	–
S5014J	Jun 30	2 d	Jejunum	+	–	–	–	–	–	–	–
S5014I	Jun 30	2 d	Ileum	+	–	–	–	–	–	–	–
S5014M	Jun 30	2 d	MLN	+	–	–	–	–	–	–	–
S5015P	Jun 15	2 d	GI pool	+	–	–	–	–	–	–	–
S5015J	Jun 30	2 d	Jejunum	+	–	–	–	–	–	–	–
S5015I	Jun 30	2 d	Ileum	+	–	–	–	–	–	–	–
S5015M	Jun 30	2 d	MLN	+	–	–	–	–	–	–	–
S5016	Jun 30	NA	Feces	+	–	–	–	–	–	–	–
S5017	Jun 30	2 d	Blood	+	–	–	–	–	–	–	–
S5018	Jun 30	2 d	Blood	+	–	–	–	–	–	–	–
S5019	Jun 30	2 d	Blood	+	–	–	–	–	–	–	–
S5020	Jun 30	2 d	Blood	+	–	–	–	–	–	–	–
S5021	Jun 30	2 d	Blood	+	–	–	–	–	–	–	–
F1	Jun 30	NA	Feed†	–	–	–	–	–	–	–	–
F2	Jul 13	NA	Feed‡	–	–	–	–	–	–	–	–
F3	Jul 13	NA	Feed‡	–	–	–	–	–	–	–	–
F4	Jul 13	NA	Feed‡	–	–	–	–	–	–	–	–
S5022	Jul 13	19 wk	Feces	+	–	–	–	–	–	–	–
S5023	Jul 13	19 wk	Feces	+	–	–	–	–	–	–	–
S5024	Jul 13	20 wk	Feces	+	–	–	–	–	–	–	–
S5025	Jul 13	20 wk	Feces	+	–	–	–	–	–	–	–
S5026	Jul 20	17 wk	Blood	+	–	–	–	–	–	–	–
S5027	Jul 20	17 wk	Blood	+	–	–	–	–	–	–	–
S5028	Jul 20	17 wk	Blood	+	–	–	–	–	–	–	–
S5029	Jul 20	17 wk	Blood	+	–	–	–	–	–	–	–
S5030	Jul 20	17 wk	Blood	+	–	–	–	–	–	–	–

*ID, identification; PDCoV, porcine delta coronavirus; PEDV, porcine epidemic diarrhea virus; TGEV, transmissible gastroenteritis coronavirus; RVA, rotavirus A; RVB, rotavirus B; RVC, rotavirus C; PPRSV, porcine reproductive and respiratory syndrome virus; +, p[ositive; – negative; NA, not available; GI, gastrointestinal; MLN, mesenteric lymph node. References for primers in PCR: PDCoV (*6*); PEDV (*2*); TGEV and RVA (*5*); RVB (*3*); RVC (*1*); PRRSV (*4*); circovirus (Veterinary Diagnostic Laboratory, Chulalongkorn University).
†From a sow.
‡From a finishing pig.

Technical Appendix Table 2. Characterization of 16 porcine deltacoronaviruses Thailand, 2015*

Virus	Sample	Date of collection	Pig age	Gene sequenced	GenBank accession no.
PDCoV/Swine/Thailand/S5011/2015	Jejunum	Jun 10	2 d	Whole genome	KU051641
PDCoV/Swine/Thailand/S5012/2015	Jejunum	Jun 10	2 d	S, E, M, N	KU051642
PDCoV/Swine/Thailand/S5013/2015	Feces	Jun 10	2 d	S, E, M, N	KU051643
PDCoV/Swine/Thailand/S5014J/2015	Jejunum	Jun 30	2 d	S, E, M, N	KU051644
PDCoV/Swine/Thailand/S5014I/2015	Ileum	Jun 30	2 d	S, E, M, N	KU051645
PDCoV/Swine/Thailand/S5014L/2015	MLN	Jun 30	2 d	S, E, M, N	KU051646
PDCoV/Swine/Thailand/S5015J/2015	Jejunum	Jun 30	2 d	S, E, M, N	KU051647
PDCoV/Swine/Thailand/S5015I/2015	Ileum	Jun 30	2 d	S, E, M, N	KU051648
PDCoV/Swine/Thailand/S5015L/2015	MLN	Jun 30	2 d	Whole genome	KU051649
PDCoV/Swine/Thailand/S5016/2015	Feces	Jun 30	2 d	S, E, M, N	KU051650
PDCoV/Swine/Thailand/S5018/2015	Blood	Jun 30	2 d	S, E, M, N	KU051651
PDCoV/Swine/Thailand/S5019/2015	Blood	Jun 30	2 d	S, E, M, N	KU051652
PDCoV/Swine/Thailand/S5022/2015	Feces	Jul 13	19 wk	S, E, M, N	KU051653
PDCoV/Swine/Thailand/S5023/2015	Feces	Jul 13	19 wk	S, E, M, N	KU051654
PDCoV/Swine/Thailand/S5024/2015	Feces	Jul 13	20 wk	S, E, M, N	KU051655
PDCoV/Swine/Thailand/S5025/2015	Feces	Jul 13	20 wk	S, E, M, N	KU051656

*PDCoV, porcine deltacoronavirus; MLN, mesenteric lymph node; S, spike; E, envelope; M, membrane; N, nucleocapsid.

Technical Appendix Table 3. Pairwise comparison of nucleotides and amino acids of Thai/S5011 porcine deltacoronavirus with those of reference viruses, Thailand, 2015*

	Gene, nucleotide (amino acid) identities, %							
Viruses	Whole genome	ORF1ab, 18,804 bp	S, 3,480 bp	E, 252 bp	M, 654 bp	NS6, 285 bp	N, 1,029 bp	NS7, 603 bp
China, Hong Kong†	98.03–98.43	98.14–98.57 (98.45–98.89)	95.95–96.68 (97.38–98.17)	99.19–100 (100)	98.60–99.07 (99.54–100)	97.86–98.94 (97.85–100)	97.29–97.81 (98.82–99.41)	97.63–98.32 (93.81–95.40)
United States‡	98.10–98.12	98.22–98.25 (98.47–98.55)	95.93–96.10 (97.20–97.82)	99.19–99.60 (100)	98.29–98.44 (100)	98.22–98.58 (98.93)	96.88–97.09 (98.53–99.12)	97.27–97.62 (92.74–93.81)
South Korea§	98.10	98.23 (98.57)	96.10 (97.73)	99.60 (100)	98.28 (100)	98.58 (98.93)	96.99 (99.12)	97.46 (93.28)
Thailand¶	99.98	99.99 (99.97)	99.97–100 (99.91–100)	100 (100)	100 (100)	100 (100)	100 (100)	100 (100)

*Gene sizes for comparison are based on isolate HKU15–155. ORF, open reading frame; S, spike; E, envelope; M, membrane; NS, nonstructural; N, nucleocapsid.
†China/AH2004/2004, HKU/15–44/2009, HKU/15–155/2010, China/S27/2012, China/HB2014/2014, China/JS2014/2014, China/JXNI2/2015.
‡USA/IA8734/2014, USA/IL121/2014, USA/IL136/2014, USA/MI8977/2014, USA/OH137/2014, USA/OH1987/2014.
§KOR/KNU14–04/2014.
¶Thailand/S5015L/2015, Thailand/S5012/2015, Thailand/S5013/2015, Thailand/S5014J/2015, Thailand/S5014I2015, Thailand/S5014L/2015, Thailand/S5015J2015, Thailand/S5015I2015, Thailand/S5016/2015, Thailand/S5018/2015, Thailand/S5019/2015, Thailand/S5022/2015, Thailand/S50232015, Thailand/S50242015, Thailand/S5025/2015.

Technical Appendix Table 4. Genetic analysis of nucleotide sequences of porcine deltacoronaviruses from Thailand and viruses from 3 other countries, 2015*

Country, virus	GenBank accession no.	Year isolated	Genome size, bp†	5′-UTR, 3-nt deletion at position 116–118	5′-UTR, 1-nt deletion at position 302	ORF1a, 6-nt deletion at position 1737-1742	ORF1a, 9-nt deletion at position 2808–2816	S gene, 3-nt insertion at position 19473–19474	3′-UTR, 3- or 4-nt insertion at position 25043–25044	3′-UTR, 1-nt deletion at position 25258	Ref
China											
HKU/15–44/2009	JQ065042	2009	25,421	No	No	No	No	ATT	GTT	T	(*12*)
HKU/15–155/2010‡	JQ065043	2010	25,416	No	No	No	No	No	No	No	(*12*)
China/S27/2012	KT266822	2012	25,404	No	No	AGTTTG	GAGCCAG TC	No	GTT	No	(*13*)
China/JXNI2/2015§	KR131621	2015	25,419	No	No	No	No	No	TT	No	(*14*)
United States											
USA/IA8734/2014¶	KJ567050	2014	25,422	No	No	No	No	AAT	GTT	No	(*15*)
South Korea											
KOR/KNU14–04/2014	KM820765	2014	25,422	No	No	No	No	AAT	GTT	No	(*16*)
Thailand											
Thailand/5011/2015#	NA	2015	25,404	TCT	A	AGTTTG	GAGCCAG TC	AAT	CTCT	No	This study
Thailand/5013/2015**	NA	2015	S, E, M, N genes	NA	NA	NA	NA	AAT	CTCT	NA	This study

*The reference virus was HKU15–155. UTR, untranslated region; ORF, open reading frame; Ref, reference; S, spike; E, envelope; M, membrane; N, nucleocapsid, NA, not available.
†Genome size does not include the polyA tail.
‡HKU/15–155/2010; China/AH2004/2004.
§China/JXNI2/2015, China/HB2014/2014, China/JS2014/2014.
¶USA/IA8734/2014, USA/IL121/2014, USA/IL136/2014, USA/MI8977/2014, USA/OH137/2014, USA/OH1987/2014.
#Thailand/5011/2015, Thailand/5015L/2015, Thailand/5012/2015, Thailand/5014L/2015, Thailand/5022/2015.
**Thailand/5013/2015, Thailand/5014J/2015, Thailand/5014I2015, Thailand/5015J2015, Thailand/5015I2015, Thailand/5016/2015, Thailand/5018/2015, Thailand/5019/2015, Thailand/50232015, Thailand/50242015, Thailand/5025/2015.

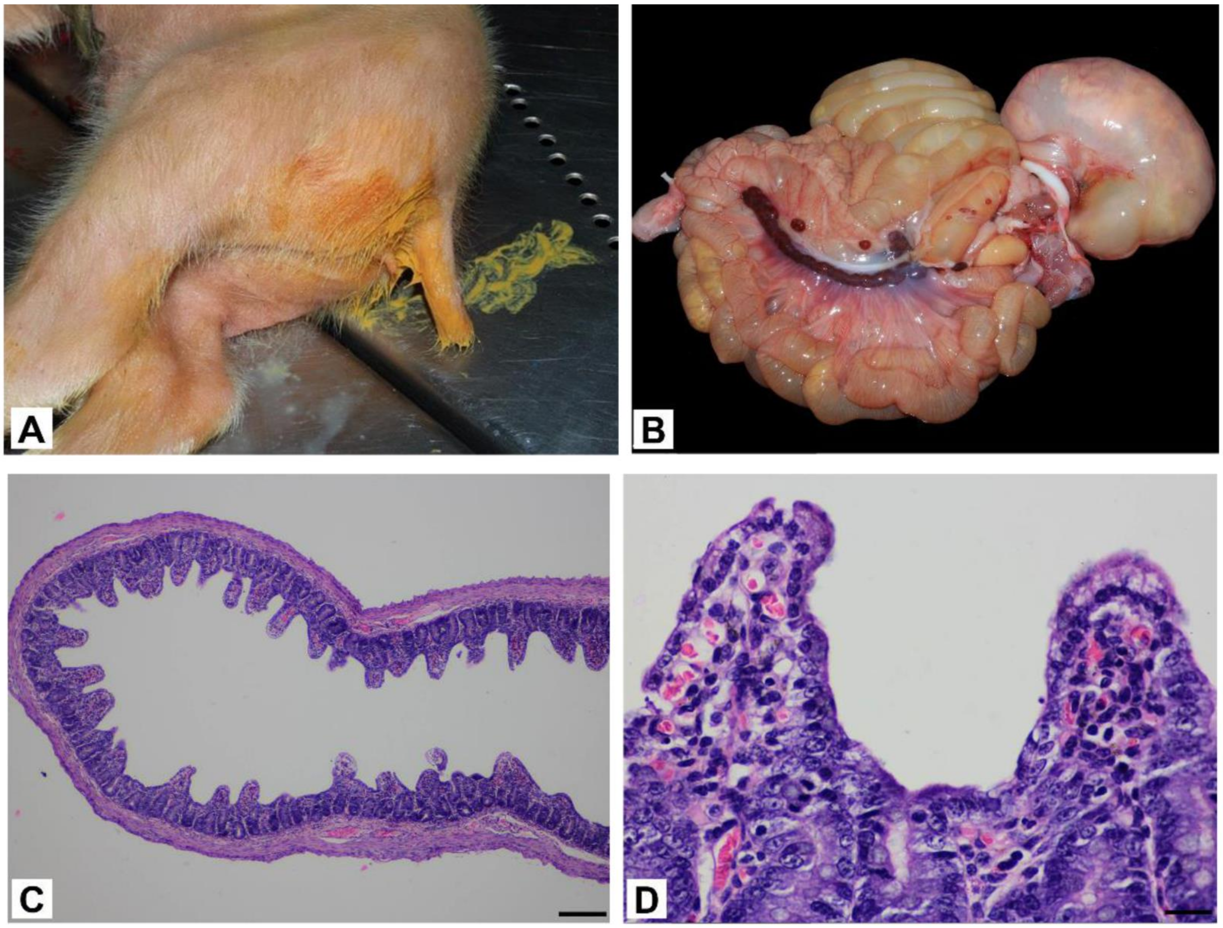

Technical Appendix Figure 1. Analysis of pigs infected with porcine deltacoronavirus, Thailand, 2015. A) Gross findings of emaciated piglet showing yellow pasty feces. B) Curdled milk in gastric lumen and thin intestinal wall containing watery content and curdled milk. Milk veins were absent. C) Histopathologic analysis showing shortened and occasionally fused villi. Scale bar = 400 μm. D) Histopathologic analysis showing attenuated and vacuolated cytoplasm of enterocytes. Scale bar = 40 μm.

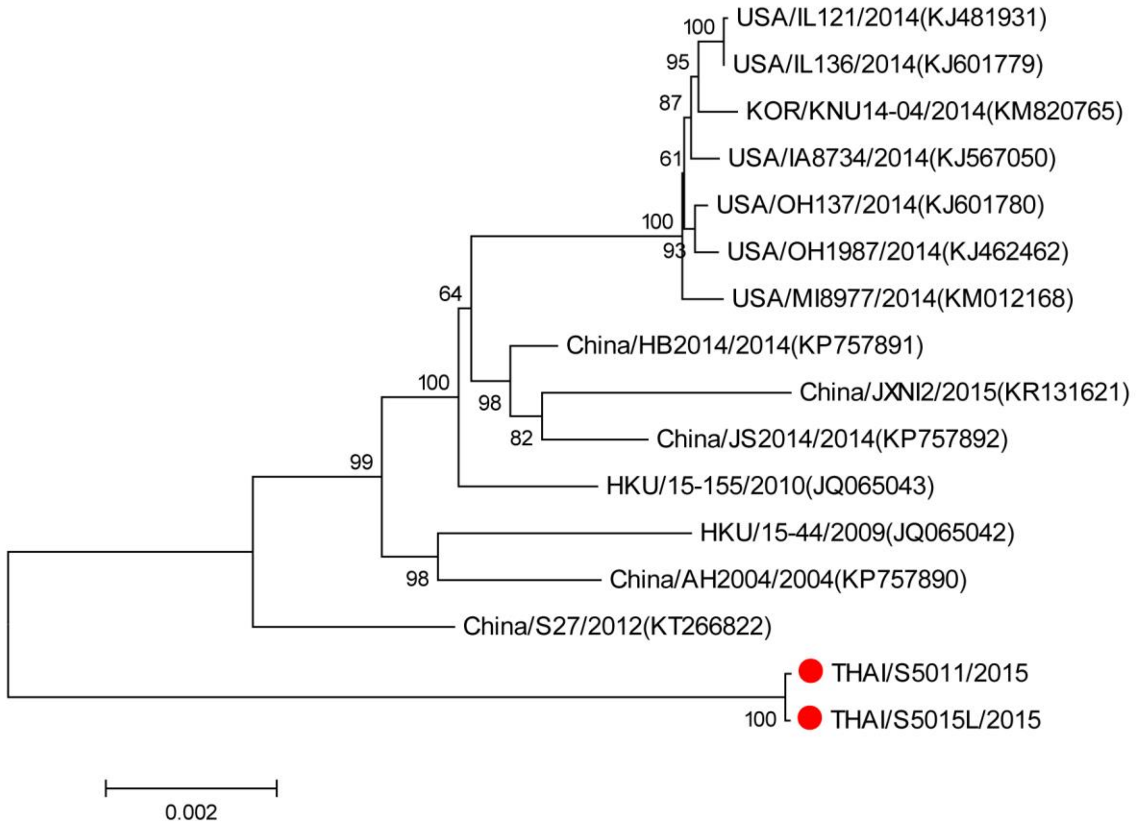

Technical Appendix Figure 2. Phylogenetic analysis open reading frame 1a/b of porcine deltacoronaviruses, Thailand, 2015. Red circles indicate strains isolated in this study. The tree was constructed by using MEGA version 6.06 program (http://www.megasoftware.net/) with the neighbor-joining algorithm and bootstrap analysis with 1,000 replications. Numbers along branches are bootstrap values. Scale bar indicates nucleotide substitutions per site.
